# Platelet-derived growth factor D expression in adrenal cells is modulated by corticosteroids: putative role in adrenal suppression

**DOI:** 10.1038/s41390-022-02094-9

**Published:** 2022-05-14

**Authors:** Christopher M. Parry, Li F. Chan, Daniel F. Carr, Daniel B. Hawcutt

**Affiliations:** 1 NIHR Alder Hey Clinical Research Facility, Liverpool, UK; 2 Department of Women’s and Children’s Health, Institute of Life Course and Medical Sciences, University of Liverpool, Liverpool, UK; 3 Centre for Endocrinology, Queen Mary University of London, London, UK; 4 Department of Pharmacology and Therapeutics, Institute of Molecular, Systems and Integrative Biology, University of Liverpool, Liverpool, UK

## Abstract

**Background:**

Adrenal suppression is a clinically concerning side effect of inhaled corticosteroid (ICS) treatment in patients with asthma. Increased susceptibility to ICS-induced adrenal suppression has previously been identified in those with the rs591118 polymorphism in platelet-derived growth factor D (*PDGFD*). The mechanism underpinning this relationship is not known.

**Methods:**

H295R cells were genotyped for rs591118 using a validated Taqman PCR allelic discrimination assay. H295R cell viability was determined after treatment with beclometasone and fluticasone (range 0–330 μM). Cortisol was measured in cell culture medium using competitive enzyme immunoassay.

**Results:**

PDGFD protein expression in H295R cells was confirmed using Western blotting. When ACTH and forskolin were added to H295R cells, a reduction in PDGFD expression was seen, which was then restored by incubation with prochloraz, a known inhibitor of steroidogenesis. A dose-dependent, decrease in PDGFD expression was observed with beclometasone (over a 24 h incubation period) but not with beclometasone incubations beyond 24 h nor with fluticasone (at 24 or 48 h).

**Conclusions:**

H295R cells express PDGFD protein, which can be modulated by incubation with steroidogenesis agonists and antagonists and additionally with exogenous beclometasone.

**Impact:**

- PDGFD is expressed in the human adrenal cell line, H295R, and expression can be modulated by beclometasone as well as agonists/antagonists of steroidogenesis.
- This builds on previous research that identified a SNP in *PDGFD* (rs591118) as an independent risk factor for adrenal suppression in adults and children with obstructive airway disease treated with inhaled corticosteroids.
- First in vitro experiments to support a link between the PDGF and cortisol production pathways, supporting the hypothesis that *PDGFD* variants can affect an individual’s sensitivity to corticosteroid-induced adrenal suppression.

## Introduction

Inhaled corticosteroids (ICS) are recommended by national and international guidelines as maintenance therapy for asthma in children and adults.^1,2^ While ICS for asthma are generally both effective and well tolerated, some patients can still experience systemic side effects. Among children and young people with asthma, adrenal suppression (AS) has been identified as being of particular clinical concern.^3^ While biochemical evidence of AS has been shown in up to 40% of children with asthma using ICS,^4^ symptomatic cases occur less frequently but can be clinically serious.^5^ AS has a highly variable clinical presentation, ranging from vague clinical signs, e.g. fatigue, nausea, abdominal pain, and growth suppression, to the classical signs of hyperpigmentation, orthostatic hypotension, hypoglycaemia, and even coma/death.^6^ The Pharmacogenomics in Childhood Asthma consortia have undertaken a prioritisation exercise to establish which adverse drug reactions (ADRs) should be the focus of research. For corticosteroids, AS was the top ranked ADR, highlighting its importance.^7^

While the dose of ICS used has an effect on the peak cortisol measured in children using ICS, it is not clinically significant.^4^ Children who take any dose of ICS may develop AS, and the dose–response relationship between total corticosteroid cumulative dose and peak cortisol is such that, with each increase of 200 mcg/day of a beclomethasone dipropionate equivalent, the peak cortisol only decreases by 0.73 nM.^4^ More recently, pharmacogenomic studies have identified that the rs591118 polymorphism in platelet-derived growth factor D (*PDGFD*) increases the risk of ICS-induced AS in both children and young people with asthma and, independently, in older adults with chronic obstructive pulmonary disease (COPD).^8^

## *PDGFD*

*PDGFD* is one of a family of four genes (A–D). *PDGFD* has not been associated with airway remodelling^9^ nor with the development and severity of asthma and COPD. Polymorphisms in *PDGFD* have also not been identified as affecting efficacy responses to corticosteroid treatment.^10^ Unlike the other PDGF isoforms, *PDGFD* is not thought to be located in any known pathways related to adrenal function, but there is evidence supporting its biological plausibility. PDGF receptors are required in the development of steroid-producing cells in multiple organs, including the testis, ovary, and adrenal cortex;^11^ *PDGFD* is highly expressed in human adrenal gland;^12^ expression of *PDGFD* has been negatively correlated with cortisol secretion in adrenocortical adenomas.^13^ However, the biological mechanisms through which steroids, adrenal cells, and *PDGFD* interact are not clear.

We hypothesised that adrenal PDGFD plays a direct role in cortisol production and subsequent AS after the treatment with glucocorticoids.

Several human adrenal cell lines have previously been described.^14^ Of those, the H295R adrenocortical carcinoma cell line is the most established and used human cell line to model adrenal function.^15^ H295R cells are more akin to zonally undifferentiated human foetal adrenal cells that, unlike other cell lines described, retain their ability to produce adrenal steroid hormones and are responsive to agonists including angiotensin II and adrenocorticotropic hormone (ACTH).^14^ This makes H295R cells a useful in vitro tool for modelling steroidogenic pathways and processes.^16–19^

In this work, we therefore utilised H295R cells to establish the role of *PDGFD* in adrenal function.

## Materials and methods

### Chemicals

Forskolin, prochloraz, fluticasone propionate, and beclomethasone dipropionate were obtained from Sigma-Aldrich (Gillingham, UK). Synacthen® (tetracosactide acetate) was obtained from Mallinckrodt Pharmaceuticals.

### H295R cell culture

H295R adrenocortical tumour cells [ATCC CRL-2128, Middlesex, UK] were cultured in 75 cm^2^ flasks containing 10 mL supplemented media at 37 °C and 5% CO_2_ atmosphere. Cells were grown in Dulbecco modified Eagle medium/F12-Ham (1:1) + GlutaMAX-I (ThermoFisher Scientific #10565018, Oxford, UK), supplemented with 5% Nu-Serum (Corning #355100, Flintshire, UK), penicillin/streptomycin, and insulin–transferrin–selenium. Cells were passaged once they reached 70–80% confluency. For experiments, cells were plated in 6-well plates and plated at 800,000 per well and incubated at 37 °C for 24 h. One day before the experiment, cells were changed to serum-free medium. The next morning, cells were treated in the same serum-free medium for the indicated times.

### Cell viability

Cell viability was assessed by 3-(4,5-dimethylthiazol-2-yl)-2,5-diphenyltetrazolium bromide (MTT) assay. Cells were plated at a density of 25,000 cells per well in a 96-well plate before changing to serum-free medium and incubation at 37 °C for further 24 h. Cells were then incubated with test reagents in quadruplicates. After 24 h, MTT solution (1 mg/ml final concentration) was added to each well and incubated for 1 h at 37 °C. In all, 100 µL solubilisation solution (10% w/v sodium dodecyl sulfate in 0.01 M HCl) was added to each well before agitating for 1 h at room temperature. Absorbance was measured at 570 nm.

### Cortisol quantification

Cortisol was measured in cell culture medium (tenfold dilution) using a competitive enzyme immunoassay kit, according to the manufacturer’s instructions (R&D Systems #KGE008B, Abingdon, UK).

### Protein extraction and Western blot

Whole-cell lysate protein concentration was determined using Bio-Rad DC Protein Assay (#5000112, Watford, UK). For Western analysis, equal amounts of protein samples were mixed with Laemmli loading buffer and boiled at 95 °C for 5 min. The reaction mix was separated on a 10% stacking gel/3% resolving gel and transferred to nitrocellulose membranes (Geneflow #B3-0010, Lichfield, UK). Membranes were incubated with primary PDGFD antibody (mouse, 1:200; Santa Cruz Biotechnology, #SC-137031) at 4 °C overnight followed by incubation with the secondary antibody (mouse, 1:5000; Cell Signaling Technology, #7076) for 1 h at room temperature. Signals were detected using chemiluminescence (Bio-Rad, #1705060, Watford, UK) and visualised using a Chemi-Doc imaging system (Bio-Rad). Glyceraldehyde 3-phosphate dehydrogenase (GAPDH) protein levels were used as a loading control. Once washed, membranes were incubated with primary GAPDH antibody (rabbit, 1:200; R&D Systems, #2275-PC-100) for 1 h at room temperature followed by incubation with the secondary antibody (rabbit, 1:5000; R&D systems, #HAF008) for 1 h at room temperature.

### DNA extraction and genotyping of H295R cells

H295R cells from both sources were subjected to DNA extraction and genotyping. Cells were thawed from frozen and centrifuged to form a cell pellet, discarding the cell supernatant. Cells were then lysed by the addition of 500 μL TRI reagent (Merck, #93289, Gillingham, UK) followed by 100 μL chloroform. The resulting mixture was then spun at 13,000 × *g* for 15 min at 4 °C. The upper aqueous phase (containing RNA) was discarded and the interphase DNA-containing layer was removed and added to 300 μL of 100% ethanol. After being left to stand for 5 min at room temperature (RT), the pellet was resuspended and washed (2×) in 0.1 M sodium citrate in 10% ethanol, centrifuging at 14,000 × *g* for 5 min at 4 °C between washes. The pellet was then resuspended in 75% ethanol and left to incubate for 20 min at RT. After removal of the ethanol the DNA pellet was left to air dry for 5 min at RT. The DNA pellet was then resolubilized in 30 μL distilled water. DNA concentration was determined using a NanoDrop spectrophotometer at 260/280 nm according to the manufacturer’s instructions.

Genotyping of H295R cells was undertaken using a validated Taqman allelic discrimination assay for rs591118 C_1951874_10 (Thermo Fisher Scientific, Paisley, UK) as described previously.^8^ rs591118 is an intronic variant in the *PDGFD* gene locus causing a A > G substitution (chr11:104095993 (GRCh38.p13).^20^

### Data processing and statistical analysis

All data were expressed as means, unless stated otherwise, and standard deviations of all data points for that concentration. Statistically significant (*p* < 0.05) differences were determined using Student’s *t* test.

## Results

### Cell viability

**Fig. 1:**
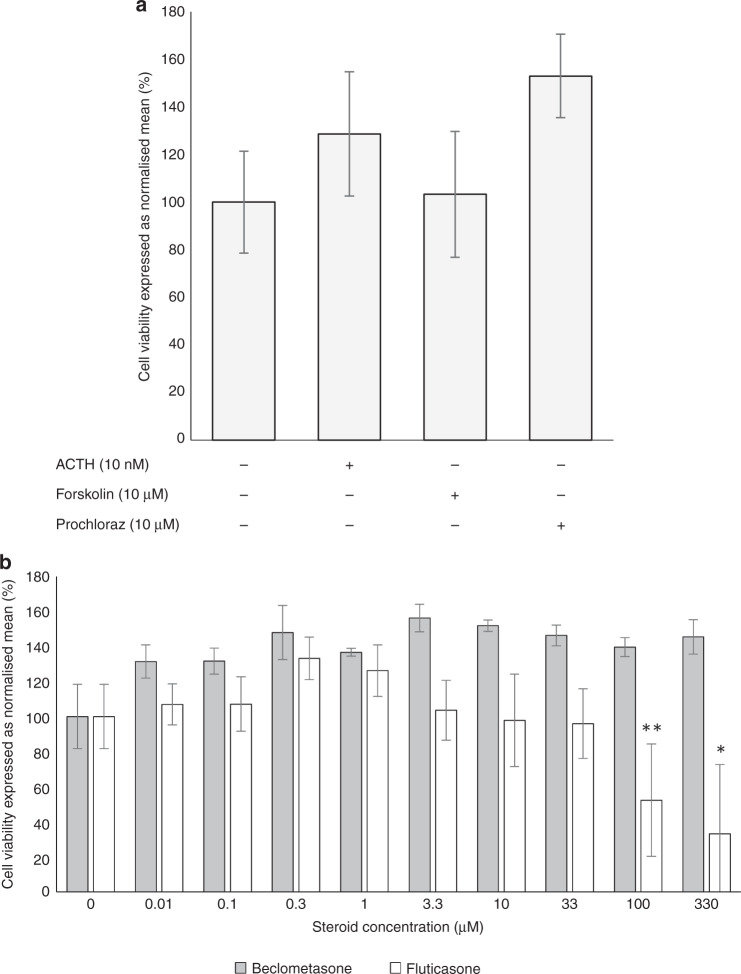
Cell viability of H295R cells as determined using MTT assay when incubated with (**a**) ACTH, forskolin and prochloraz and (**b**) beclometasone diproprionate and fluticasone propionate. Error bars represent standard deviation; *n* = 4, *p < 0.05, **p < 0.01.

Incubation with maximum concentrations of ACTH, forskolin, prochloraz, and beclometasone did not demonstrate any negative effect on H295R cell viability (Fig. 1a, b) as shown in previous works.^21^ Incubation with fluticasone, however, demonstrated a drop off in cell viability at concentrations >33 μM (Fig. 1b). Therefore, subsequent steroid incubation experiments were done with a maximum fluticasone concentration of 33 μM.

### Genotype at rs591118

H295R cells were homozygous for the major allele of rs591118 (G/G), in keeping with the literature.^20^

### Expression of PDGFD in H295R cells is regulated by ACTH, forskolin, and prochloraz

**Fig. 2:**
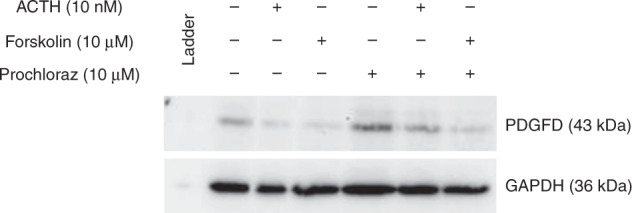
Effect of ACTH, forskolin and prochloraz on PDGFD protein levels in H295R cells. Once treated for 24 h, protein extracts were harvested for Western blot. Twenty micrograms of cell extract were then subjected to 10% SDS-PAGE.

The presence of PDGFD protein in H295R cells was successfully established using Western blotting. The independent addition of both ACTH and forskolin resulted in downregulation of PDGFD expression. This was restored by incubation with prochloraz, a known inhibitor of steroidogenesis (Fig. 2).

**Fig. 3:**
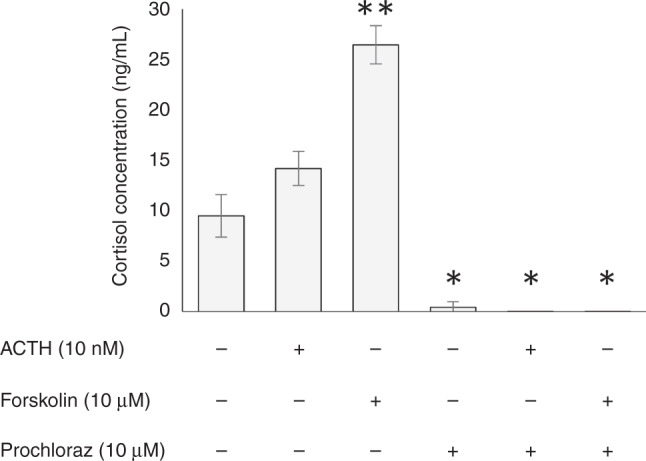
Changes in H295R cortisol production when treated with ACTH, forskolin and prochloraz, alone and in combination. Cortisol concentrations are presented as mean ±  standard deviation; *n* = 2, *p < 0.05, **p < 0.01.

H295R cells were also responsive to both ACTH and forskolin with a relative increase in cortisol of 1.5- and 2.8-fold, respectively (Fig. 3). This was in keeping with previous works in the literature.^22,23^ Addition of prochloraz resulted in complete loss of detectable cortisol production that could not be restored by the addition of ACTH or forskolin.

### H295R cell PDGFD expression with exogenous corticosteroid treatment

**Fig. 4:**
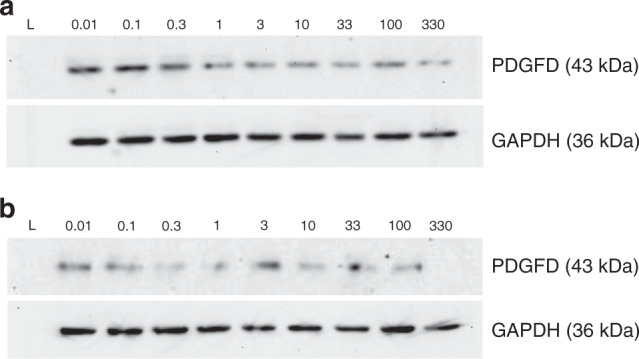
Effect of beclometasone diproprionate on PDGFD expression in H295R cells. **a** Incubated with beclometasone for 24 h, (**b**) incubated with beclometasone for 48 h. Lane keys: (L) Protein ladder (#161-035, Bio-Rad, Watford, UK), 0.01 to 330 μM beclometasone concentration.

**Fig. 5:**
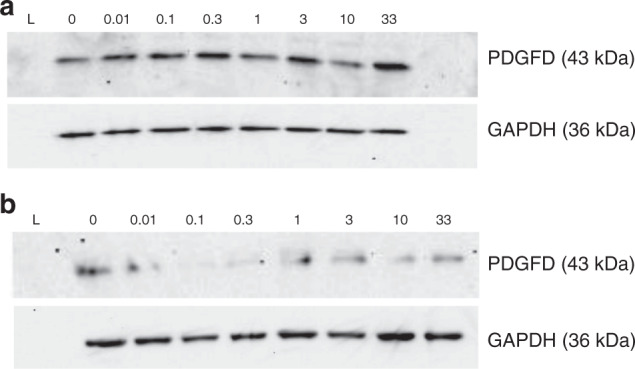
Effect of fluticasone proprionate on PDGFD expression in H295R cells. **a** Incubated with fluticasone for 24 h, (**b**) incubated with fluticasone for 48 h. Lane keys: (L) Protein ladder (#161-035, Bio-Rad, Watford, UK), 0 to 33 μM fluticasone concentration.

H295R cells were incubated with increasing concentrations of beclometasone (0–330 μM) and fluticasone (0–33 μM) for 24 or 48 h. Figure 4a shows a beclometasone dose-dependent decrease in PDGFD expression, at 24 h. However, this pattern is not clearly seen for beclometasone incubations at 48 h nor with incubations with fluticasone (Figs. 4b and 5a, b). Nevertheless, when compared with the 24 h incubations, there does appear to be a decrease in PDGFD expression (at all concentrations) when incubated with beclometasone or fluticasone for 48 h.

Cortisol concentrations in the cell supernatant were also measured during these experiments. After discussion with the manufacturer, it was confirmed that the kit had not been specifically tested for cross-reactivity between cortisol and beclometasone or fluticasone. Therefore, beclometasone and fluticasone were prepared at the same concentrations used in the experiments above and assayed for cross-reactivity. Beclometasone showed <0.1% cross-reactivity and fluticasone demonstrated a maximum of 2.5% cross-reactivity at the highest concentration of fluticasone.

Both steroids show a similar pattern in that the cortisol response, across all concentrations, is fairly flat when incubated with steroid for 24 h (Supplementary Fig. 1), with no significant difference compared to baseline control (*p* > 0.05). However, when H295R cells were incubated for 48 h, the cortisol levels show an upwards trajectory, particularly significant for beclometasone (*p* < 0.05). These results are not dissimilar to previous works looking at the effects of ICS on the plasma cortisol concentrations of healthy adults over a period of hours/short days.^24,25^

## Discussion

This is the first study to explore the mechanistic relationships concerning the observed pharmacogenomic relationship between PGFD polymorphism and susceptibility to AS. The mechanisms underpinning the link between the hypothalamic–pituitary–adrenal (HPA) axis and the PDGF signalling pathway are not currently described. Therefore, this work represents the first step in exploring links between these two signalling pathways. We have demonstrated that human adrenal cell line, H295R, expresses PDGFD protein, which can be modulated by incubation with steroidogenesis agonists and antagonists and with certain exogenous steroids (beclometasone). We have also demonstrated that the genotype of H295R cells (from both Dr Li Chan and ATCC) at single-nucleotide polymorphism (SNP) rs591118 is GG, in keeping with the most common allele frequency.^20^ Further works will be required to determine whether attenuation, and overexpression, of PDGFD using small interfering RNA methodologies changes the effects on cortisol secretion when H295R cells are exposed to exogenous steroids.

While considerable additional work is still required, this work provides a valuable step towards development of personalised medicine in childhood asthma. If individuals with specific PDGFD variants are at increased risk of steroid-induced AS, personalising therapy using either steroid-sparing agents or more intensive clinical monitoring of their HPA axis would maximise the safety of treatment.

The main limitation of this work is that the findings seen by incubation of H295R cells with beclometasone could not be confidently replicated with fluticasone. At the chemical level, fluticasone is highly lipophilic and has a much greater affinity for the glucocorticoid receptor than beclometasone^26^ resulting in longer retention times in lung tissues.^27^ Added together, this results in a doubling of the relative potency of fluticasone (vs. beclometasone) and therefore a lower dose requirement when used in vivo.^28^ These factors may help to explain the clinical observation that fluticasone causes greater AS than more hydrophilic ICS at equivalent dosing ranges.^29^ This may also explain our findings that, at higher concentrations, fluticasone was toxic to H295R cells and so longer, lower-dose experiments may be required including in cells displaying each of the three genotypes (AA, AG, GG) at the rs591118 SNP.

A second limitation relates to the nature of adrenal cell lines, which are few in number and of the immortalised lines, only H295R are noted to be responsive to ACTH signalling.^14^ One future direction would involve CRISPR knockout experiments with PDGFD to further understand the interactions, but careful selection of cell lines will be required before such future experiments.

Finally, the HPA axis is a complex system with multiple levels and negative feedback loops. While further work with other exogenous steroids will be required, it may be that the changes noted in PDGFD levels are reflective of changes at the level of the hypothalamus and pituitary gland, rather than the adrenal gland, and that changes in PDGFD are reflective more of the effects of exogenous steroids higher up in the HPA axis, which would be consistent with our current understanding of steroid-induced, tertiary AS.^30^ Future work will need to consider the pituitary and hypothalamus as well as adrenal roles in this putative relationship.

Overall, this work expands upon the clinical pharmacogenomic findings previously published^8^ and provides a platform upon which further detailed exploratory work can be carried out to identify the exact relationship.

## Supplementary information

Supplementary Figure

## Data Availability

The data sets generated during and/or analysed during the current study are available from the corresponding author on reasonable request.
