# Supplementary material for: Platelet-derived growth factor D expression in adrenal cells is modulated by corticosteroids: putative role in adrenal suppression
Source: Pediatr Res. 2022 May 14;93(1):97–101. doi: 10.1038/s41390-022-02094-9 (PMC9876782; doi:10.1038/s41390-022-02094-9)
Supplement: Supplementary file 1 — Supplementary Figure [file 41390_2022_2094_MOESM1_ESM.pdf]

(a)

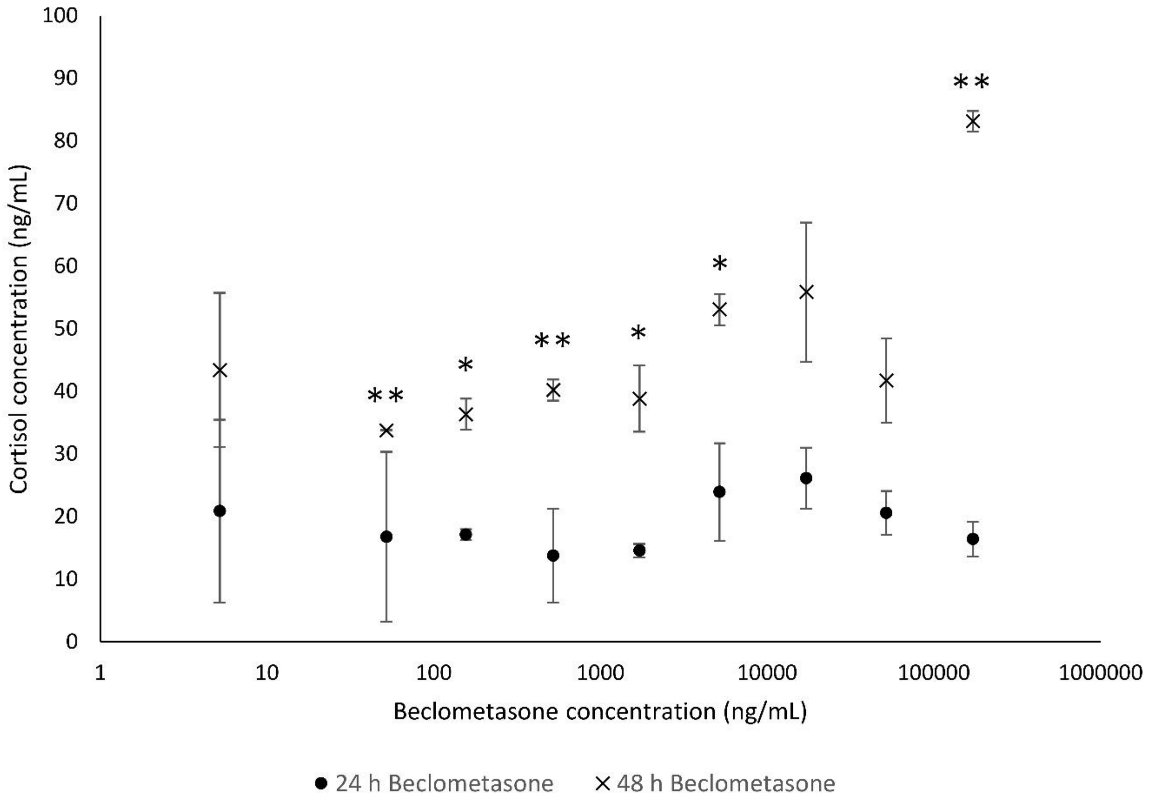

100
90
80
70
60
50
40
30
20
10
0
Cortisol concentration (ng/mL)
1
10
100
1000
10000
100000
1000000
Beclometasone concentration (ng/mL)
**
*
**
*
*
**
24 h Beclometasone
48 h Beclometasone

(b)

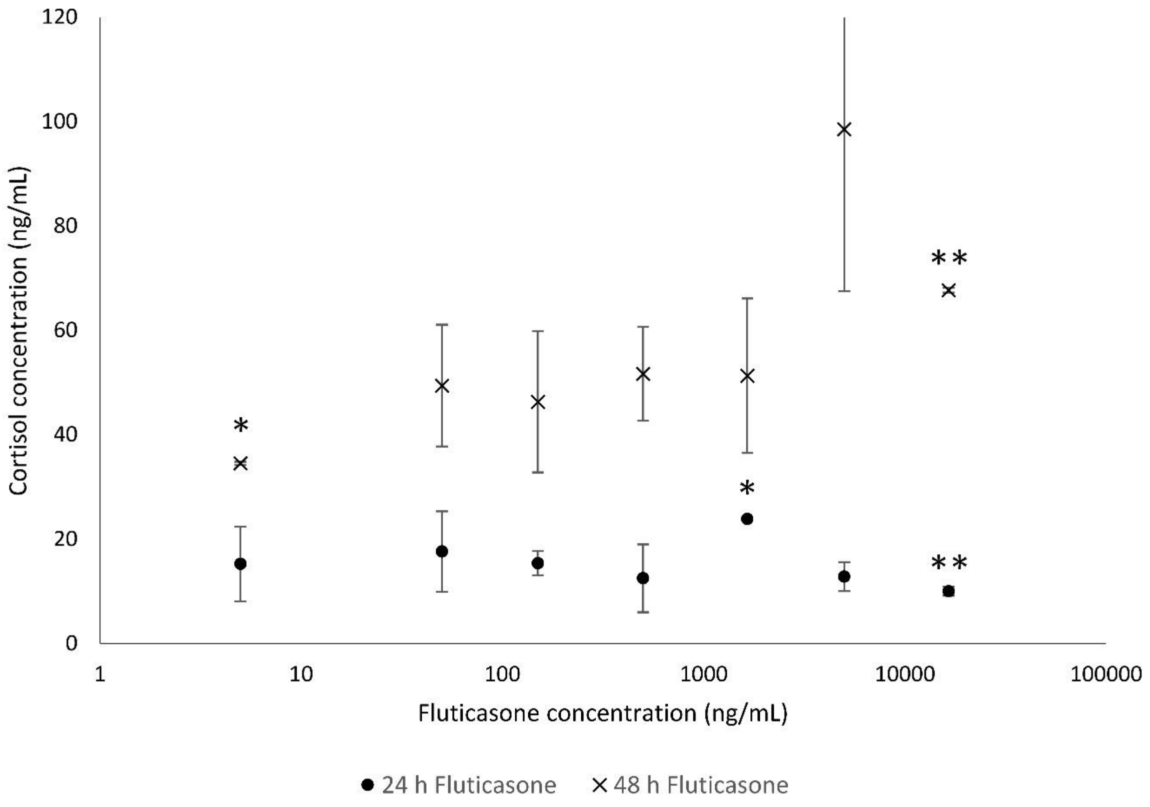

Supplementary Figure 1. Cortisol levels (ng/mL) in H295R cell supernatant after being treated with (a) beclometasone for 24 h (●) and 48 h (×) and (b) fluticasone. Error bars represent standard deviation; n=2-3. *p < 0.05. **p < 0.01. ***p < 0.001.
